# Niche partitioning and the storage effect facilitate coexistence in an amphibian community

**DOI:** 10.1002/ece3.10629

**Published:** 2023-10-18

**Authors:** George C. Brooks, Nicholas M. Caruso, Houston C. Chandler, Carola A. Haas

**Affiliations:** ^1^ Department of Fish and Wildlife Conservation Virginia Tech Blacksburg Virginia USA; ^2^ The Orianne Society Tiger Georgia USA

**Keywords:** dominance, ephemeral wetlands, evenness, phenology, synchrony

## Abstract

Virtually all natural community assemblages are dominated by a handful of common species. Dominant species can exert negative impacts on biodiversity through competitive exclusion, and thus there is a strong incentive to understand imbalances in community composition, changes in dominance hierarchies through time, and mechanisms of coexistence. Pond‐breeding amphibians that utilize ephemeral wetlands provide an excellent opportunity to evaluate theoretical predictions of community composition in stochastic environments. One of the most striking features of pond‐breeding amphibians is the marked stochastic fluctuations in abundance across years. Given strong theoretical and empirical links between evenness and biomass, one would expect community evenness to change from year to year. Moreover, if different species exhibit different boom‐and‐bust reproductive cycles, then a storage effect may help to explain why one species does not outcompete all others. Here, we explore the interplay between biotic and abiotic conditions in shaping amphibian communities at two ephemeral wetlands on Eglin Air Force Base, Florida. We document consistent community composition over 6 years of monitoring, resulting from a lack of species turnover and similar responses of all community members to environmental conditions. The similar dynamics of species argues against a storage effect as the sole mechanism for coexistence and instead points to niche partitioning as a more important factor. In support of this conclusion, we show that the degree of synchrony in breeding migrations only correlates with environmental conditions within species, not between species. The lack of pattern seen between species implies that individuals are somewhat constrained in the timing of breeding migrations, perhaps owing in part to competition with other community members. We hope that our work reinvigorates interest in amphibian communities and highlights ephemeral wetlands as model systems to study community dynamics in stochastic environments.

## Graphical abstract

We explored the interplay between biotic and abiotic conditions in shaping amphibian communities at two ephemeral wetlands on Eglin Air Force Base, Florida. We documented similar dynamics across community members arguing against a storage effect as the sole mechanism for coexistence and instead points to niche partitioning playing a critical role in reducing interspecific competition. We hope that our work reinvigorates interest in amphibian communities and highlights ephemeral wetlands as model systems to study community dynamics in stochastic environments.

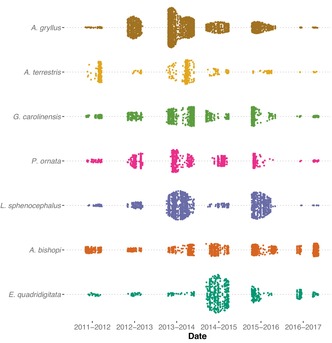

## 1 INTRODUCTION

In stochastic environments, differences in species' responses to environmental conditions provide a mechanism for coexistence through a storage effect (Angert et al., 2009; Johnson & Hastings, 2022; Warner & Chesson, 1985). Storage effects arise through variable levels of interannual recruitment that generates asynchronous dynamics across community members, facilitating coexistence (Johnson & Hastings, 2022; Warner & Chesson, 1985). If boom recruitment years for one species do not necessarily reflect boom recruitment years for others, intraspecific competition largely overshadows interspecific competition, thus limiting the capacity for competitive exclusion (Angert et al., 2009; Chesson & Warner, 1981; Hardin, 1960; Johnson & Hastings, 2022). If instead population cycles are synchronous across community members, interspecific competition will be prohibitively high in boom years, and an alternative mechanism for coexistence must be operating (Angert et al., 2009; Johnson & Hastings, 2022). For communities composed of species whose recruitment success relies on the same environmental conditions, competition may be alleviated, and coexistence facilitated, by niche partitioning (Hardin, 1960). Differences in breeding phenology, habitat proclivities, and trophic position have all been shown to reduce competition between co‐occurring species (Denoël & Joly, 2001; Vignoli et al., 2017). The degree to which species can partition niche space in stochastic environments, however, remains unclear. Moreover, the relative importance of niche partitioning compared to the storage effect in promoting community stability is seldom explicitly investigated (but see Crowder et al., 2011).

Pond‐breeding amphibians that utilize ephemeral wetlands provide an excellent opportunity to evaluate theoretical predictions of community composition in stochastic environments (Anderson et al., 2017; Snodgrass et al., 2000). Pond‐breeding amphibians typically exhibit marked fluctuations in abundance across years (Berven, 1990; Pechmann et al., 1991; Semlitsch, 1983, 1987; Semlitsch et al., 1996, 2014; Taylor et al., 2006; Whiteman & Wissinger, 2005). Furthermore, asynchronous population dynamics appear to be the norm (Mouillot & Wilson, 2002; Semlitsch et al., 1996; Trenham et al., 2003; Weiher & Keddy, 1999; Werner et al., 2007), with year‐to‐year variability in the relative abundance of community members indicating a potential storage effect (Chesson & Warner, 1981). Niche partitioning is also common in pond‐breeding amphibian communities (e.g., Denoël & Joly, 2001; Sinsch et al., 2012; Toft, 1985). Given that community members must time their breeding migrations to coincide with periods of inundation for successful reproduction to take place, interspecific competition is expected to be high (Anderson et al., 2017; Godbold & Solan, 2009; Wohlgemuth et al., 2016). Wet years, however, provide a degree of flexibility in the timing of breeding and thus permit individuals to stagger their arrivals to reduce competition. By studying both fluctuations in abundance and the timing of breeding activities, it is possible to discern the relative importance of storage effects and niche partitioning in shaping amphibian communities.

Here, we explore the interplay between biotic and abiotic conditions in shaping amphibian communities at two ephemeral wetlands on Eglin Air Force Base, Florida. Using 6 years of drift fence data, our objectives were to (1) calculate community evenness and relate it to variation in biomass through time, (2) describe the timing of movements for the seven most commonly encountered amphibian species (*Acris gryllus*, *Anaxyrus terrestris*, *Gastrophryne carolinensis*, *Pseudacris ornata*, *Rana sphenocephala*, *Ambystoma bishopi*, *Eurycea quadridigitata*), and (3) relate the degree of synchrony in movements to environmental conditions. We predicted that, as evidence for a storage effect, both the total biomass of all species and the biomass of individual species (relative to other species) would fluctuate across years, resulting in changes in community ordering and the degree of evenness. If instead niche partitioning is driving coexistence, we would predict that the timing of movements between species would be consistently staggered in time to reduce temporal overlap, but that dry years would result in a greater overlap in phenology due to the limited availability of breeding habitat. We discuss our findings in relation to the conservation of pond‐breeding amphibians and our understanding of community dynamics in stochastic environments.

## 2 METHODS

### 2.1 Field methods

To monitor community composition through time, we completely encircled two wetlands on Eglin Air Force Base, Florida with drift fences and funnel traps (see Erwin et al., 2016 for additional details). We constructed drift fences from 60 cm tall metal flashing buried in the sediment approximately 15–20 cm. We placed funnel traps (85 × 20 cm in size) flush with the fence and ground at approximately 10 m intervals on both sides of the fence (Gibbons & Semlitsch, 1981; Korfel et al., 2010). While drift fences have certain biases associated with them (e.g., for certain sized individuals or against certain species that can climb over fences), they have been shown to achieve a comprehensive sample of amphibian communities in wetland systems under a variety of conditions (Gunzburger, 2007; Semlitsch et al., 1996; Todd et al., 2007). The climate at our study sites is characterized by a hot summer season running from May to October, and a cool winter season running from November to April. We ran drift fences discontinuously from 2011 to 2017, typically opening fences in late October or early November with the onset of fall rains that fill ephemeral wetlands and trigger amphibian breeding (Table 1). Study wetlands typically hold water through the following spring and dry up with rising summer temperatures. We closed drift fences in March if wetlands were already dry with no amphibian reproduction likely to occur, or May if wetlands held water later into the year. We typically checked traps multiple times per night over the entire period. For each captured amphibian, we recorded the species, date, and time of capture. Following processing, we released all individuals on the opposite side of the fence from which they were caught. For all analysis related to the timing of arrivals, we only used data from individuals encountered on the outside of the fence.

**TABLE 1:** Dates of drift fence operation.

Season	Dates	Nights	Total abundance	Daily abundance	Total biomass (g)	Daily biomass (g)	Evenness
2011–12	10/31–03/23	150	822	5	7920	53	0.65
2012–13	10/29–03/12	135	1560	11	7502	56	0.76
2013–14	10/06–05/31*	255	4144	16	28,731	113	0.64
2014–15	09/13–03/29	195	2116	9	10,428	53	0.82
2015–16	10/12–05/04*	165	2453	14	20,769	126	0.52
2016–17	11/08–05/24*	100	809	8	4059	41	0.54

*Note:* Ponds were completely encircled, and fences were operated continuously between the dates shown apart from those marked with asterisks where fences were run continuously until March and then run sporadically (3–4 times per week) until fence closure in May. For all years, fences were temporarily closed for 1–2 weeks in late December or early January due to staffing shortages. Nights reflect the total number of sampling nights that were conducted between the start and end dates each year. Daily abundance and daily biomass reflect the total abundance and total biomass respectively, divided by the number of sampling nights in that year.

We installed monitoring wells in the deepest part of each wetland on August 18, 2011, which consisted of a 3.8 cm diameter screened PVC pipe 1 m below ground. At the bottom of each well, we placed a HOBO U20 pressure transducer (Onset Computer Corporation, Bourne, MA) that recorded barometric pressure at 15‐minute intervals. To convert barometric pressure readings into water levels, we installed a dry well less than 500 m from both wetlands using the same methods and materials and placed a pressure transducer in the well head space (McLaughlin & Cohen, 2011). We classified hydroperiod each year as the longest consecutive number of days each wetland held water (i.e., water level > 0) between the months of October and May.

### 2.2 Statistical analyses

For each year of the study, we calculated Pielou's evenness (Pielou, 1966) using the counts of adults arriving at breeding wetlands for all amphibian species. We chose Pielou's evenness scores as they benefit from not being conflated with species richness, and thus allow direct comparison of estimates across studies or systems (Magurran, 1988; Pielou, 1975; Simpson, 1949; Smith & Wilson, 1996).

For statistical tractability (i.e., adequate sample size), all additional analyses only included seven of the eight most encountered amphibians. Eastern newts (*Notophthalmus viridescens*) were excluded from subsequent analysis because they are not thought of as winter breeders and have a unique life history that is not typically associated with ephemeral wetlands (Petranka, 1998). To test theoretical predictions of the relationship between community evenness and biomass, we first made crude estimates of biomass by multiplying the number of each species by average mass values taken from the literature (Anura: *Acris gryllus* = 0.45 g, *Anaxyrus terrestris* = 19 g, *Gastrophryne carolinensis* = 1 g, *Pseudacris ornata* = 5 g, *Rana sphenocephala* = 22 g, Caudata: *Ambystoma bishopi* = 6 g, *Eurycea quadridigitata* = 0.75 g; Blem et al., 1978; Conant & Collins, 1998; Petranka, 1998; Wygoda, 1984). We acknowledge that this omits variability in mass across individuals (e.g., adults vs. metamorphs) within populations; however, the bias in our total biomass estimates introduced through this methodology should not impact the qualitative nature of our results because the inherent bias will be similar across years. We scaled annual biomass estimates by the number of trapping days in each season (Table 1), and then performed linear regression of evenness over time as a function of scaled annual biomass.

We then sought to describe the pattern of movements over the course of the study. Owing to the periodic nature of our data, we employed circular regression to compare within and between season arrival times for the seven most encountered amphibians (Cremers & Klugkist, 2018; Pewsey et al., 2013). Day of year was transformed into radians to create a continuous, circular variable that can be used as the response in a circular regression. We constructed a Bayesian model that included species and year as predictors, and their interaction, and compared this to simpler models. Each model was run for 1000 iterations, with the first 500 draws discarded as burn‐in. Models were compared using the deviance information criterion (DIC) and the Watanabe–Akaike information criterion (WAIC), and the significance of variables was indicated by whether the 95% highest posterior density intervals included zero (Cremers, 2021; Gelman et al., 2014).

To then relate differences in the timing of movements to environmental covariates, we calculated several summary metrics. Specifically, we recorded the dates when 50%, 75%, and 90% of the sampled population had arrived and used these point estimates to conduct further analyses. This approach reduces biases related to sample size and extreme values that would be present in an analysis of first arrival dates (Mills, 2005; Van Buskirk et al., 2009). To quantify breeding synchrony within species, we calculated the number of days between the 50% and 90% thresholds for each species in each year and constructed a generalized linear mixed model with hydroperiod as a predictor and species as a random effect. To quantify breeding synchrony across species within years, we calculated the difference between the earliest arriving species and the latest arriving species each year and performed a simple linear regression modeling the difference in days as a function of hydroperiod. Confidence intervals and *p*‐values for the effect of hydroperiod were calculated using Wald's t‐distribution approximation. All analyses were performed in R using the *circular*, *bpnreg*, and *lme4* packages (Agostinelli & Lund, 2022; Bates et al., 2015; Cremers, 2021; R Core Team, 2023).

## 3 RESULTS

Over the duration of the study, 12,335 amphibians were encountered at drift fences. The amphibian community showed drastic interannual variability in the total abundance of all species (Table 1; Figure S1). The amphibian community was dominated by five anuran species; southern cricket frogs (*Acris gryllus*, 21% of captures), southern leopard frogs (*Rana sphenocephala*, 12% of captures), eastern narrow‐mouthed toads (*Gastrophryne carolinensis*, 11% of captures), ornate chorus frogs (*Pseudacris ornata*, 9% of captures), and southern toads (*Anaxyrus terrestris*, 4% of captures), and three caudate species; reticulated flatwoods salamanders (*Ambystoma bishopi*, 14% of captures), eastern newts (*Notophthalmus viridescens*, 6% of captures), and dwarf salamanders (*Eurycea quadridigitata*, 5% of captures). Although all these species were caught frequently at drift fences throughout the sampling season (which targeted winter breeding amphibians), eastern narrow‐mouthed toads, and to a lesser extent the southern cricket frogs and southern toads, are more typically considered spring or summer breeders (Petranka, 1998; Wells, 2010). Nine other amphibian species were encountered during sampling but were captured in such low numbers (<10 individuals, e.g., *Pseudacris nigrita*) or have ecologies associated with different waterbodies (e.g., *Amphiuma means*) such that we think they were transient individuals that never used the study sites for breeding. Thus, these nine species were excluded from all analyses.

**FIGURE 1:**
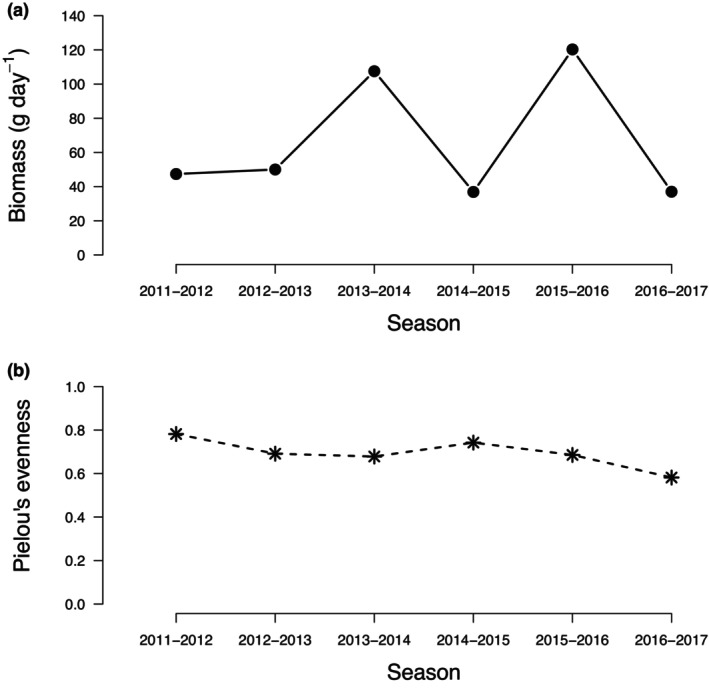
Relationship between evenness and biomass for each year of drift fence monitoring.

**FIGURE 2:**
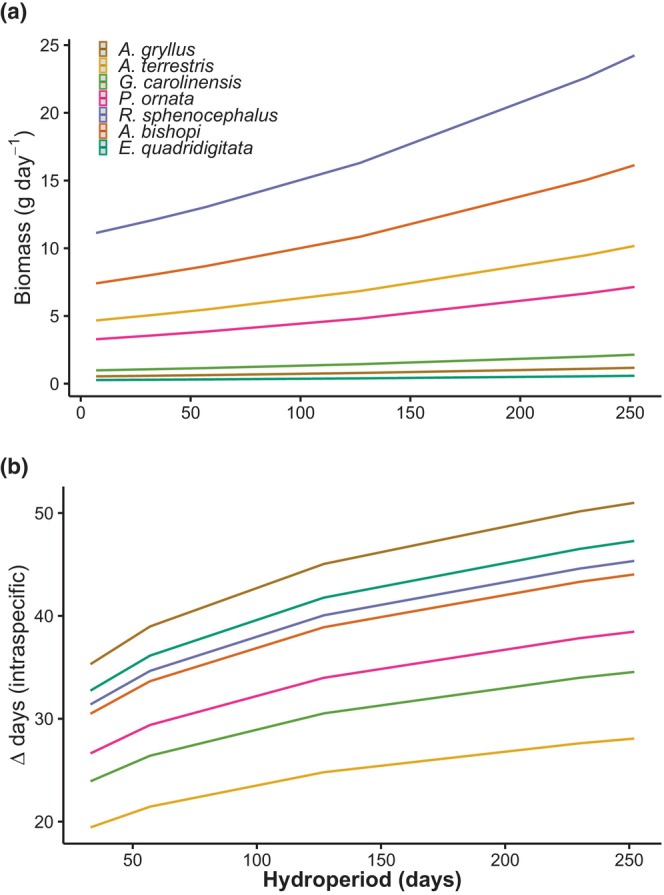
Relationship between hydroperiod and (a) daily biomass, (b) intraspecific synchrony in arrival times (i.e., the time between the earliest and latest arrivals for a given species).

Daily biomass estimates ranged from 41 g in the 2016–2017 season to 126 g in the 2015–2016 season (Table 1; Figure 1a). In all but the first and last years of the study, southern leopard frogs were the dominant species (Table 2). In the first year, southern toads were the dominant species and in the last year reticulated flatwoods salamanders were dominant (Table 2). Across all encountered amphibian species, community evenness scores remained relatively consistent, ranging from 0.58 in the 2016–2017 season to 0.78 in the 2011–2012 season (Table 1; Figure 1b). Annual fluctuations in biomass and dominance hierarchies, however, showed no predictable relationship with community evenness (Figure 1). Instead, we found evidence that total annual biomass was related to environmental conditions. Specifically, interannual variation in both the timing and amount of precipitation resulted in variable wetland hydroperiods, and years with longer hydroperiods corresponded with the highest values of daily biomass ($R_{c}^{2}$= .71, *p* = .040; Figure 2a). This pattern, however, of more animals in years with longer hydroperiods, varied by species; when considered in isolation, southern cricket frogs, eastern narrow‐mouthed toads, and dwarf salamanders showed no relationship between biomass and hydroperiod across the study duration (Figure 2a).

**TABLE 2:** Relative abundance of amphibians as a percentage of the total annual biomass.

Species	2011–2012	2012–2013	2013–2014	2014–2015	2015–2016	2016–2017
*A. gryllus*	0.2%	4.3%	2.5%	2.8%	0.9%	0.4%
*A. terrestris*	**59.4%**	9.3%	18.8%	12.7%	3.5%	6.2%
*G. carolinensis*	1.2%	3.0%	2.3%	2.3%	2.2%	3.0%
*P. ornata*	4.2%	20.2%	7.9%	12.9%	6.4%	5.3%
*R. sphenocephalus*	15.2%	**51.5%**	**60.7%**	**33.4%**	**73.3%**	6.5%
*A. bishopi*	19.4%	11.4%	7.7%	32.1%	13.2%	**76.9%**
*E. quadridigitata*	0.3%	0.3%	0.1%	0.4%	0.4%	1.8%

*Note:* Numbers highlighted in bold represent the dominant species in each breeding season.

**FIGURE 3:**
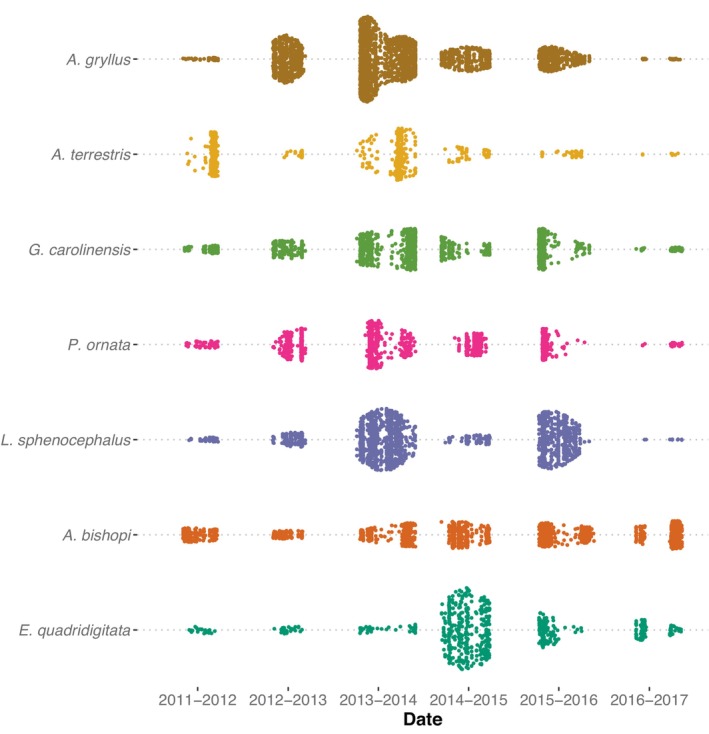
Abundance through time at two drift fenced wetlands for the five most commonly encountered anuran species (*Rana sphenocephala*, *Pseudacris ornata*, *Gastrophryne carolinensis*, *Anaxyrus terrestris*, *Acris gryllus*) and the two most commonly encountered salamander species (*Eurycea quadridigitata*, *Ambystoma bishopi*). Each dot represents a single capture event and the spread of points from the central dotted line for each species is proportional to the density of captures on that date.

**FIGURE 4:**
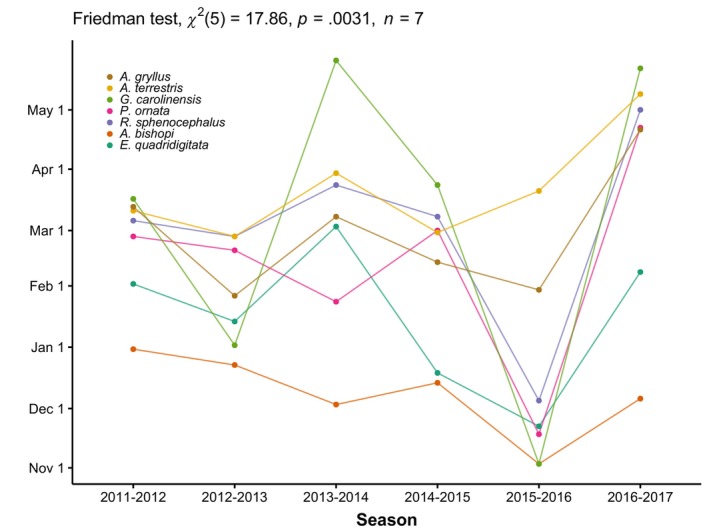
Timing of annual breeding migrations for seven of the most commonly encountered amphibians at drift fences surrounding two wetlands on Eglin Air Force Base, Florida. Point estimates reflect when 75% of the adult population of that species has arrived at breeding wetlands. Annual variability in migration timing outweighs variability in arrival times between species in a given year (*χ* ^2^ = 17.86, *p* = .0031).

The best supported model for timing of movements included species, year, and their interaction (Table 3). Across all species and years, the earliest mean movement date was 2 October and the latest was 30 May. Pairwise comparisons revealed evidence for differences in species' arrival times regardless of year (Table S1). In contrast, differences between years were only apparent when accounting for species‐specific responses to environmental conditions (Tables S2 and S3). Phenological patterns did appear to have a taxonomic basis, with salamander populations arriving earlier than anurans in most years (Figures 3 and 4). Eastern narrow‐mouthed toads were the last species to arrive in 4 of the 6 years (Figure 4). Southern toads and eastern narrow‐mouthed toads displayed the most synchronous breeding migrations, with the average time between 50% movement and 90% of animal movements being 22 and 27 days, respectively. Southern cricket frogs exhibited the most asynchronous migrations, with an average of 62 days between 50% and 90% of movements. Furthermore, the degree of synchrony in movement was partially related to environmental conditions (Figure 2b). Arrival times of individuals within each species were more synchronized in years with shorter hydroperiods ($R_{c}^{2}$= .13, *p* = .023). In contrast, synchrony in arrival times between species showed no relationship with hydroperiod ($R_{c}^{2}$= .04, *p* = .67).

**TABLE 3:** Comparison of circular regression models describing the timing of amphibian breeding migrations as a function of species and year.

Model	DIC	ΔDIC	WAIC	ΔWAIC	$\mathrm{WAI}C_{w}$
~Species×year	39,467	0	39,468	0	1.00
~Species+year	39,743	276	39,743	275	0.00
~Year	39,905	438	39,905	437	0.00
~Species	40,045	578	40,045	577	0.00
~1	40,258	791	40,258	790	0.00

*Note*: Day of year (converted to radians) was used as the response. Models were compared using the deviance information criterion (DIC) and the Watanabe–Akaike information criterion (WAIC). WAIC_*w*_ represent the model weights.

## 4 DISCUSSION

Here, we describe community dynamics of amphibians breeding in two ephemeral wetlands in the southeastern United States. Similar to the results of other studies on amphibian communities (Pechmann et al., 1991; Semlitsch et al., 1996, 2014), abundance and biomass fluctuated dramatically across years, with peaks in biomass corresponding to conditions conducive to successful reproduction. Despite such annual variability in biomass, community evenness remained relatively constant through time, perhaps due to ecological differences that reduce interspecific competition. In support of this idea, species showed consistent differences in the timing of movements across a range of environmental conditions. In contrast, the degree of synchrony in movements within species was strongly correlated with environmental conditions. Our results shed new light on the structuring of amphibian communities that breed in ephemeral wetlands and the forces that promote coexistence.

In contrast to previous long‐term studies of wetland communities (e.g., Semlitsch et al., 1996; Werner et al., 2007), we found no evidence for species turnover (in our seven focal species) at either of our study sites over the course of the study. Local extinctions, skipped breeding years, and a lack of site fidelity can all drive rates of species turnover at amphibian breeding wetlands (Hernández‐Ordóñez et al., 2019). Community composition at these wetlands, however, appears largely fixed, suggesting that the phenomena are absent or negligible in this system (Hernández‐Ordóñez et al., 2019; Werner et al., 2007). Species persistence at ephemeral wetlands has been shown to be most strongly correlated with regional, as opposed to local, population size (da Silva et al., 2011; Hernández‐Ordóñez et al., 2019; Werner et al., 2007), indicating a large regional population of each species encountered at these wetlands. We also found no evidence for constant turnover in the dominance structure (defined by biomass of each species) of community members. In some systems, the identity of the dominant species exerts the strongest influence on biomass production, and thus the identity of the dominant species indirectly impacts community composition (Jones & Magurran, 2018; Massaccesi et al., 2015). In other instances, however, it is the relative productivity of the dominant species, rather than the identity of that species per se, that controls community structure and evenness (Jones & Magurran, 2018; Poggio & Ghersa, 2011). Our results suggest that dominance structure and the identity of dominant species are being tightly regulated, which will strongly influence ecosystem functioning and carry implications for population assessments and the monitoring of threatened and endangered species (Gotelli et al., 2017; Winfree et al., 2015).

In contrast to expectations of how evenness will change across an abundance gradient, we find remarkable consistency in community evenness, despite dramatic fluctuations in the abundance of all seven focal amphibians. As a result, we conclude that although successful reproduction may occur in different years for different species (Figure 3), the storage effect on its own cannot explain coexistence. Many studies have documented more even communities when all community members are rare (Bazzaz, 1975; Bock et al., 2007; Estrada‐Villegas et al., 2012; Latham et al., 1994; Trenham & Shaffer, 2005; Weiher & Keddy, 1999; Whittaker, 1965), and theory predicts that such patterns can emerge through real biological processes or simply by the underlying distribution of abundances (Drobner et al., 1998; Weiher & Keddy, 1999). We speculate that consistency in evenness in our study likely results from niche and/or fitness differences between community members (Akatov et al., 2018; Chalcraft et al., 2009; Hanlin et al., 2000; HilleRisLambers et al., 2012; Wilsey & Potvin, 2000). For example, flatwoods salamanders deposit eggs in dry wetland basins prior to their inundation (Anderson & Williamson, 1976) and as such, are typically the first to arrive at breeding sites. In addition, unlike other ambystomatids (Holomuzki & Collins, 1987; Petranka, 1998; Wilbur, 1972), flatwoods salamanders do not appear to exert strong predation pressure on other amphibian larvae, potentially decoupling their population dynamics from other community members.

As predicted, populations tended to exhibit more synchronous breeding migrations in years with limited rainfall. This pattern suggests that amphibians are at least somewhat able to assess the breeding potential of a given year based on environmental cues and time their movements to maximize reproductive success (e.g., Benard & Greenwald, 2023; Brooks et al., 2019). Alternatively, some amphibians may opt to skip certain years if the chance of reproductive success is low, and the observed synchrony results merely as an artifact of fewer individuals moving in dry years (Cayuela et al., 2014, 2016). Although numerous studies have shown skipped breeding years in pond‐breeding amphibians (Cayuela et al., 2014, 2016; Pechmann et al., 1991; Trenham & Shaffer, 2005), we did not find a strong relationship between environmental conditions and abundance, suggesting that intraspecific synchrony in our study is being driven in part by concurrent movement. Future work should aim to identify the cues that amphibians use when deciding whether to attempt breeding or not.

While environmental conditions strongly influence the onset of breeding migrations in many amphibian species, observed patterns of interspecific synchrony failed to yield a simple environmental explanation. The consistent differences in arrival times between species and the synchronous fluctuations in abundance across years both imply that coexistence is predominantly facilitated by niche partitioning as opposed to the storage effect (Chesson & Warner, 1981; Johnson & Hastings, 2022; Warner & Chesson, 1985). Different phenological patterns help to reduce interspecific competition and can even allow similar species to occupy different trophic levels (Anderson et al., 2017, 2021; Vignoli et al., 2017). In amphibians specifically, communities are often split into fall and spring breeders (Wells, 2010). Here, we show that a range of breeding strategies are present among species typically grouped as fall breeders, for example, early arrival in *A. bishopi*, explosive breeding in *G. carolinensis*. Moreover, our data show that the timing of breeding is consistently staggered throughout the season. Although staggered breeding can sometimes lead to competitive exclusion through priority effects (Sredl & Collins, 1991), it significantly reduces the temporal overlap of species cohabiting breeding wetlands (Anderson et al., 2017, 2021; Toft, 1985). Moreover, such staggered breeding can facilitate niche partitioning if the larvae of the earliest breeders grow large enough such that there is no dietary overlap with the larvae of late breeders (Anderson et al., 2017, 2021; Vignoli et al., 2017).

Two artifacts of our study design limit the precision of our findings. Firstly, we did not weigh every animal encountered at the drift fences and are thus forced to use a crude estimate of biomass for each species. Accounting for the size distribution of populations would provide more reliable biomass–evenness relationships, especially given that some amphibians change in size by an order of magnitude from metamorph to adult. Secondly, the number of trap nights, and perhaps more importantly the timing of drift fence operations, was different across years. Although every effort was made to sample on nights when capture probabilities were high, we cannot rule out the possibility that big pulses of movement were missed. Correcting for sample effort each year helped to alleviate some of these potential issues, but it does not account for any taxonomic bias in movement events on nights the fences were not run. Despite these shortcomings, our results raise interesting unanswered questions concerning how individuals evaluate breeding potential each year, what are the key determinants of stability in ephemeral wetland amphibian communities, and how does such stability or lack thereof contribute to species persistence at the broader landscape scale. We hope that our work reinvigorates interest in amphibian communities and highlights ephemeral wetlands as model systems to study community dynamics in stochastic environments.

## AUTHOR CONTRIBUTIONS

**George C. Brooks:** Conceptualization (lead); data curation (equal); formal analysis (equal); methodology (equal); validation (equal); visualization (lead); writing – original draft (lead); writing – review and editing (lead). **Nicholas M. Caruso:** Conceptualization (supporting); data curation (equal); formal analysis (supporting); investigation (equal); methodology (equal); writing – review and editing (equal). **Houston C. Chandler:** Conceptualization (supporting); data curation (supporting); formal analysis (supporting); investigation (supporting); methodology (supporting); writing – review and editing (equal). **Carola A. Haas:** Conceptualization (equal); funding acquisition (lead); investigation (equal); methodology (equal); project administration (equal); resources (equal); supervision (equal); writing – review and editing (equal).

## CONFLICT OF INTEREST STATEMENT

The authors have no conflicts of interest to declare.

## Supporting information


Table S1

Table S2

Table S3

Figure S1

## Data Availability

All data and code used to perform the analysis have been archived on a public GitHub repository: https://github.com/geobro1992/synchrony.
