# Supplementary material for: Niche partitioning and the storage effect facilitate coexistence in an amphibian community
Source: Ecol Evol. 2023 Oct 18;13(10):e10629. doi: 10.1002/ece3.10629 (PMC10585123; doi:10.1002/ece3.10629)
Supplement: Supplementary file 1 — Table S1 [file ECE3-13-e10629-s004.docx]

Table S1. Post hoc pairwise comparisons for the year-only circular regression. Numbers reported reflect the difference between the posterior means and associated 95% confidence intervals. Differences where the 95% confidence interval does not overlap zero (i.e., p < 0.05) are marked with an asterisk.

| Comparison | Difference | sd |
| --- | --- | --- |
| 2011 – 2012 | 1.63 | 0.99 |
| 2011 – 2013 | **2.74*** | 1.00 |
| 2011 – 2014 | -1.79 | 1.03 |
| 2011 – 2015 | 0.90 | 1.00 |
| 2011 – 2016 | 0.04 | 1.02 |
| 2012 – 2013 | **2.22*** | 0.95 |
| 2012 – 2014 | 2.09 | 0.93 |
| 2012 – 2015 | 1.37 | 1.00 |
| 2012 – 2016 | 1.30 | 1.04 |
| 2013 – 2014 | **3.10*** | 0.87 |
| 2013 – 2015 | 1.76 | 0.99 |
| 2013 – 2016 | **2.46*** | 0.92 |
| 2014 – 2015 | 0.98 | 1.12 |
| 2014 – 2016 | -0.92 | 1.29 |
| 2015 – 2016 | 0.71 | 1.07 |
