# Supplementary material for: Niche partitioning and the storage effect facilitate coexistence in an amphibian community
Source: Ecol Evol. 2023 Oct 18;13(10):e10629. doi: 10.1002/ece3.10629 (PMC10585123; doi:10.1002/ece3.10629)
Supplement: Supplementary file 2 — Table S2 [file ECE3-13-e10629-s002.docx]

Table S2. Post hoc pairwise comparisons for the species-only circular regression. Numbers reported reflect the difference between the posterior means and associated 95% confidence intervals. Differences where the 95% confidence interval does not overlap zero (i.e., p < 0.05) are marked with an asterisk.

| Comparison | Difference | sd |
| --- | --- | --- |
| *E. quadridigitatta – A. bishopi* | **1.23*** | 0.61 |
| *E. quadridigitatta – L. sphenocephalus* | **1.35*** | 0.55 |
| *E. quadridigitatta – P. ornata* | **1.79*** | 0.53 |
| *E. quadridigitatta – G. carolinensis* | 1.08 | 0.55 |
| *E. quadridigitatta – A. terrestris* | **2.89*** | 0.53 |
| *E. quadridigitatta – A. gryllus* | **-3.04*** | 0.55 |
| *A. bishopi – L. sphenocephalus* | **1.73*** | 0.55 |
| *A. bishopi – P. ornata* | **1.93*** | 0.51 |
| *A. bishopi – G. carolinensis* | **1.59*** | 0.59 |
| *A. bishopi – A. terrestris* | **2.78*** | 0.46 |
| *A. bishopi – A. gryllus* | **2.86*** | 0.39 |
| *L. sphenocephalus – P. ornata* | **1.85*** | 0.51 |
| *L. sphenocephalus – G. carolinensis* | **1.55*** | 0.56 |
| *L. sphenocephalus – A. terrestris* | **2.63*** | 0.47 |
| *L. sphenocephalus – A. gryllus* | **2.52*** | 0.44 |
| *P. ornata – G. carolinensis* | **1.79*** | 0.52 |
| *P. ornata – A. terrestris* | **2.51*** | 0.48 |
| *P. ornata – A. gryllus* | **2.37*** | 0.47 |
| *G. carolinensis – A. terrestris* | **2.64*** | 0.47 |
| *G. carolinensis – A. gryllus* | **2.52*** | 0.43 |
| *A. terrestris – A. gryllus* | **3.01*** | 0.47 |
