# Supplementary material for: Niche partitioning and the storage effect facilitate coexistence in an amphibian community
Source: Ecol Evol. 2023 Oct 18;13(10):e10629. doi: 10.1002/ece3.10629 (PMC10585123; doi:10.1002/ece3.10629)
Supplement: Supplementary file 3 — Table S3 [file ECE3-13-e10629-s003.docx]

Table S3. Post hoc pairwise comparisons for the circular regression including species and year. Numbers reported reflect the difference between the posterior means and standard deviations (sd). Differences where the 95% confidence interval does not overlap zero (i.e., p < 0.05) are marked with an asterisk.

| Comparison | Difference | sd |
| --- | --- | --- |
| 2011 – 2012 | **1.81*** | 0.38 |
| 2011 – 2013 | **3.10*** | 0.63 |
| 2011 – 2014 | **-0.80*** | 0.38 |
| 2011 – 2015 | **1.20*** | 0.30 |
| 2011 – 2016 | 0.27 | 0.28 |
| 2012 – 2013 | **2.57*** | 0.34 |
| 2012 – 2014 | **2.42*** | 0.40 |
| 2012 – 2015 | **1.87*** | 0.32 |
| 2012 – 2016 | **1.79*** | 0.39 |
| 2013 – 2014 | **-2.84*** | 0.37 |
| 2013 – 2015 | **2.24*** | 0.36 |
| 2013 – 2016 | **2.70*** | 0.65 |
| 2014 – 2015 | **1.68*** | 0.45 |
| 2014 – 2016 | -0.35 | 1.04 |
| 2015 – 2016 | **1.27*** | 0.36 |
| *A. bishopi – L. sphenocephalus* | 0.27 | 0.60 |
| *A. bishopi – P. ornata* | **1.48*** | 0.46 |
| *A. bishopi – G. carolinensis* | 0.24 | 0.54 |
| *A. bishopi – A. terrestris* | **3.11*** | 0.32 |
| *A. bishopi – A. gryllus* | -1.15 | 0.67 |
| *L. sphenocephalus – P. ornata* | **1.13*** | 0.41 |
| *L. sphenocephalus – G. carolinensis* | 0.29 | 0.42 |
| *L. sphenocephalus – A. terrestris* | **2.93*** | 0.42 |
| *L. sphenocephalus – A. gryllus* | -0.52 | 0.58 |
| *P. ornata – G. carolinensis* | **1.05*** | 0.41 |
| *P. ornata – A. terrestris* | **2.62*** | 0.32 |
| *P. ornata – A. gryllus* | 1.46 | 1.08 |
| *G. carolinensis – A. terrestris* | **2.90*** | 0.45 |
| *G. carolinensis – A. gryllus* | -0.46 | 0.53 |
| *A. terrestris – A. gryllus* | **-2.79*** | 0.32 |
|  |  |  |
| *2011* |  |  |
| *E. quadridigitatta – A. bishopi* | 0.02 | 0.31 |
| *E. quadridigitatta – L. sphenocephalus* | 0.17 | 0.25 |
| *E. quadridigitatta – P. ornate* | **1.11*** | 0.33 |
| *E. quadridigitatta – G. carolinensis* | 0.16 | 0.25 |
| *E. quadridigitatta – A. terrestris* | **3.11*** | 0.42 |
| *E. quadridigitatta – A. gryllus* | **-0.76*** | 0.31 |
|  |  |  |
| 2012 |  |  |
| *E. quadridigitatta – A. bishopi* | **2.07*** | 0.32 |
| *E. quadridigitatta – L. sphenocephalus* | **1.74*** | 0.32 |
| *E. quadridigitatta – P. ornate* | **1.95*** | 0.30 |
| *E. quadridigitatta – G. carolinensis* | **1.67*** | 0.33 |
| *E. quadridigitatta – A. terrestris* | **2.66*** | 0.29 |
| *E. quadridigitatta – A. gryllus* | **2.40*** | 0.34 |
|  |  |  |
| 2013 |  |  |
| *E. quadridigitatta – A. bishopi* | **3.10*** | 0.32 |
| *E. quadridigitatta – L. sphenocephalus* | **2.78*** | 0.38 |
| *E. quadridigitatta – P. ornate* | **2.47*** | 0.31 |
| *E. quadridigitatta – G. carolinensis* | **2.70*** | 0.42 |
| *E. quadridigitatta – A. terrestris* | **3.12*** | 0.28 |
| *E. quadridigitatta – A. gryllus* | **-2.65*** | 0.31 |
|  |  |  |
| 2014 |  |  |
| *E. quadridigitatta – A. bishopi* | **-1.54*** | 0.56 |
| *E. quadridigitatta – L. sphenocephalus* | -0.47 | 0.52 |
| *E. quadridigitatta – P. ornate* | **1.91*** | 0.46 |
| *E. quadridigitatta – G. carolinensis* | -0.38 | 0.47 |
| *E. quadridigitatta – A. terrestris* | **-2.92*** | 0.28 |
| *E. quadridigitatta – A. gryllus* | **-1.60*** | 0.33 |
|  |  |  |
| 2015 |  |  |
| *E. quadridigitatta – A. bishopi* | **1.45*** | 0.31 |
| *E. quadridigitatta – L. sphenocephalus* | **1.21*** | 0.30 |
| *E. quadridigitatta – P. ornate* | **1.58*** | 0.30 |
| *E. quadridigitatta – G. carolinensis* | **1.15*** | 0.31 |
| *E. quadridigitatta – A. terrestris* | **2.42*** | 0.29 |
| *E. quadridigitatta – A. gryllus* | **1.42*** | 0.34 |
|  |  |  |
| 2016 |  |  |
| *E. quadridigitatta – A. bishopi* | 0.44 | 0.42 |
| *E. quadridigitatta – L. sphenocephalus* | 0.41 | 0.36 |
| *E. quadridigitatta – P. ornata* | **1.23*** | 0.36 |
| *E. quadridigitatta – G. carolinensis* | 0.38 | 0.36 |
| *E. quadridigitatta – A. terrestris* | **2.87*** | 0.34 |
| *E. quadridigitatta – A. gryllus* | -0.48 | 0.48 |
