# Supplementary material for: Niche partitioning and the storage effect facilitate coexistence in an amphibian community
Source: Ecol Evol. 2023 Oct 18;13(10):e10629. doi: 10.1002/ece3.10629 (PMC10585123; doi:10.1002/ece3.10629)
Supplement: Supplementary file 4 — Figure S1 [file ECE3-13-e10629-s001.pdf]

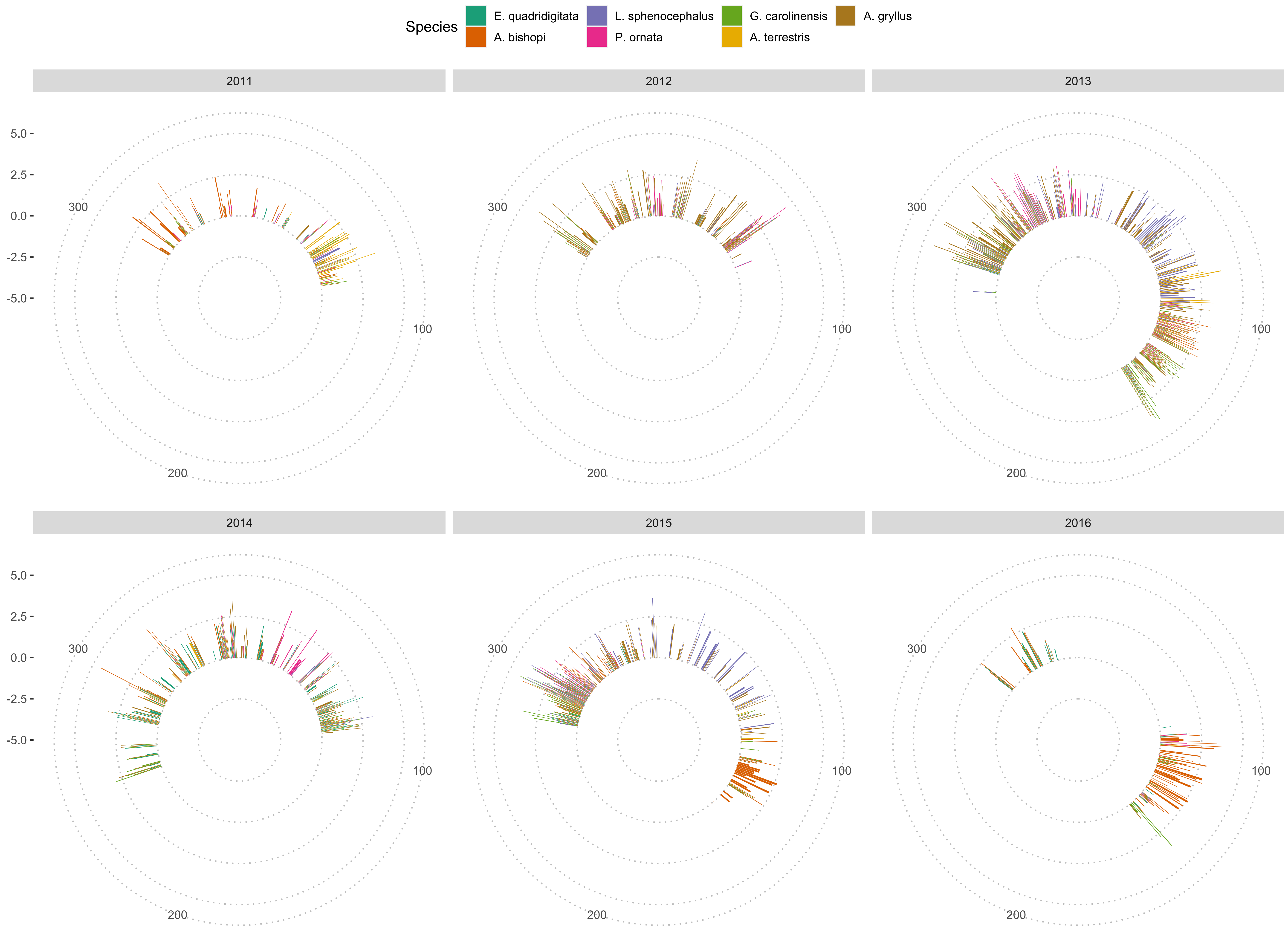

Figure S1. Radial plot of species arrival times over the six years of drift-fence monitoring.
Day of year has been converted to degrees such that January 1st equals 1 and December 31st equals 360.
Heights of bars are scaled to the number of individuals captured per day.
